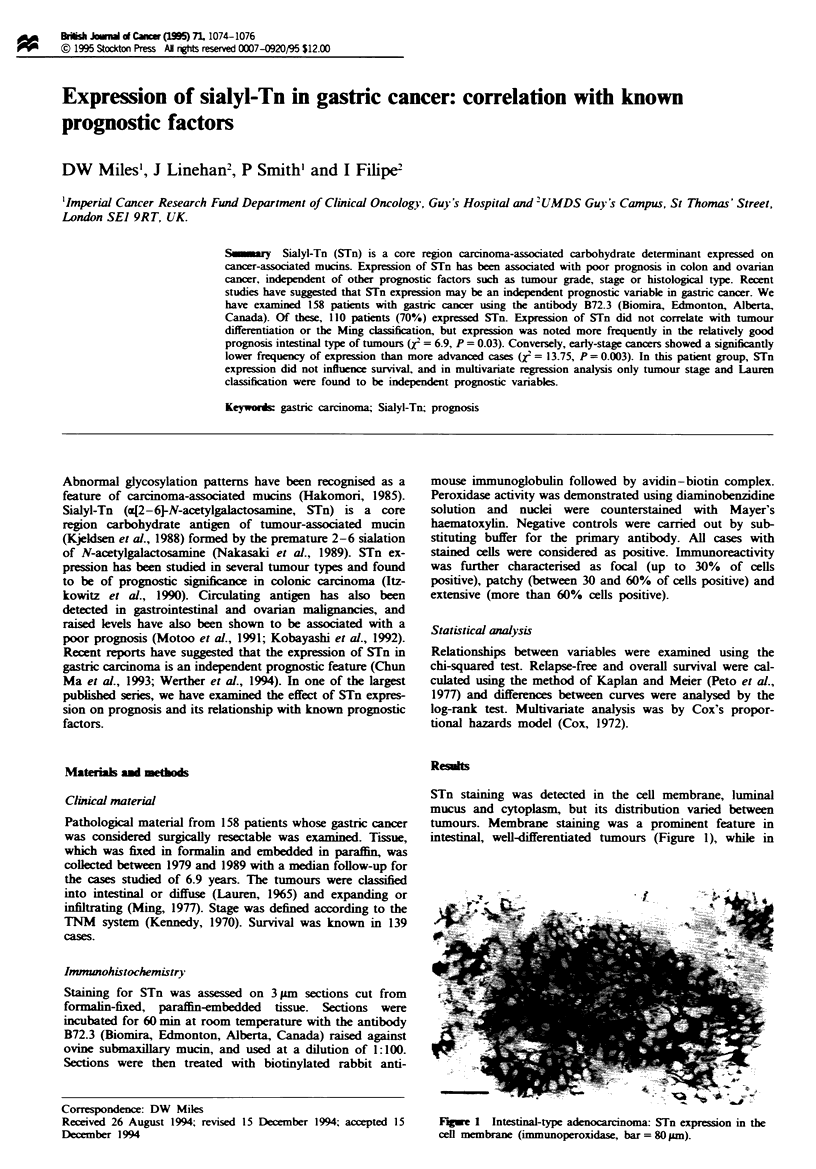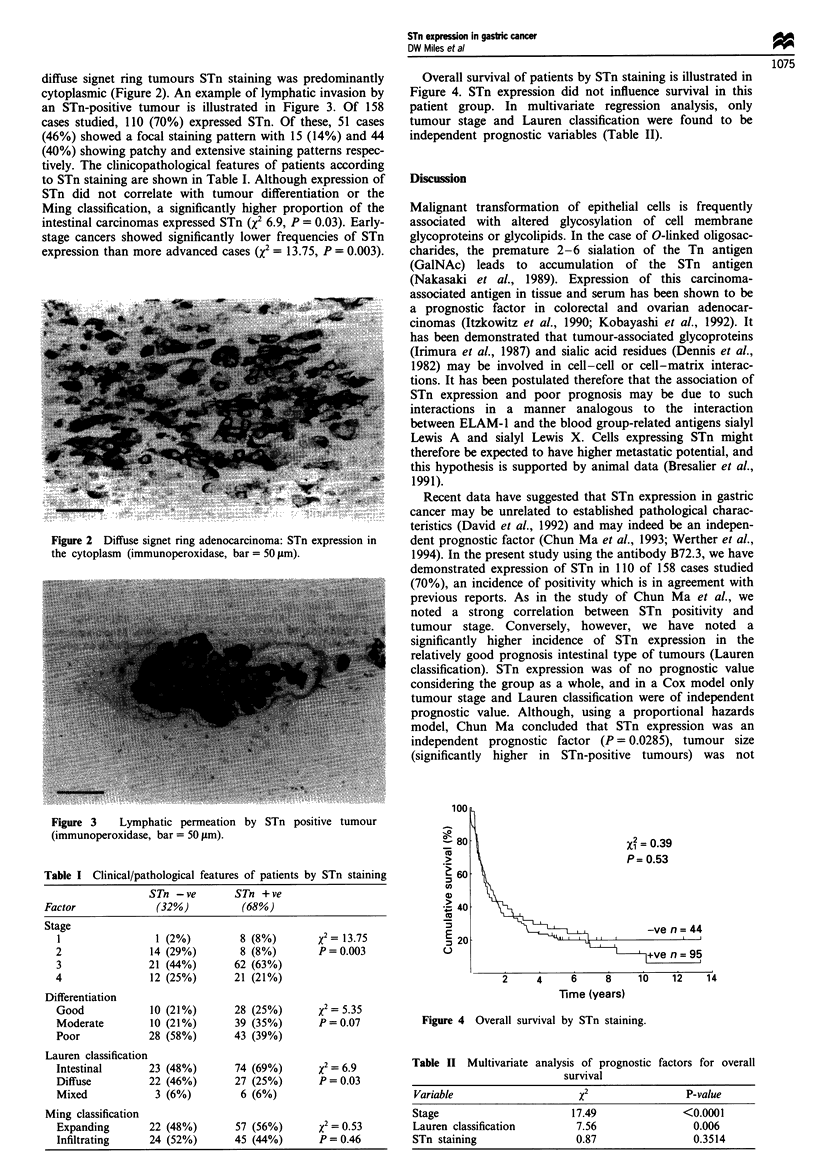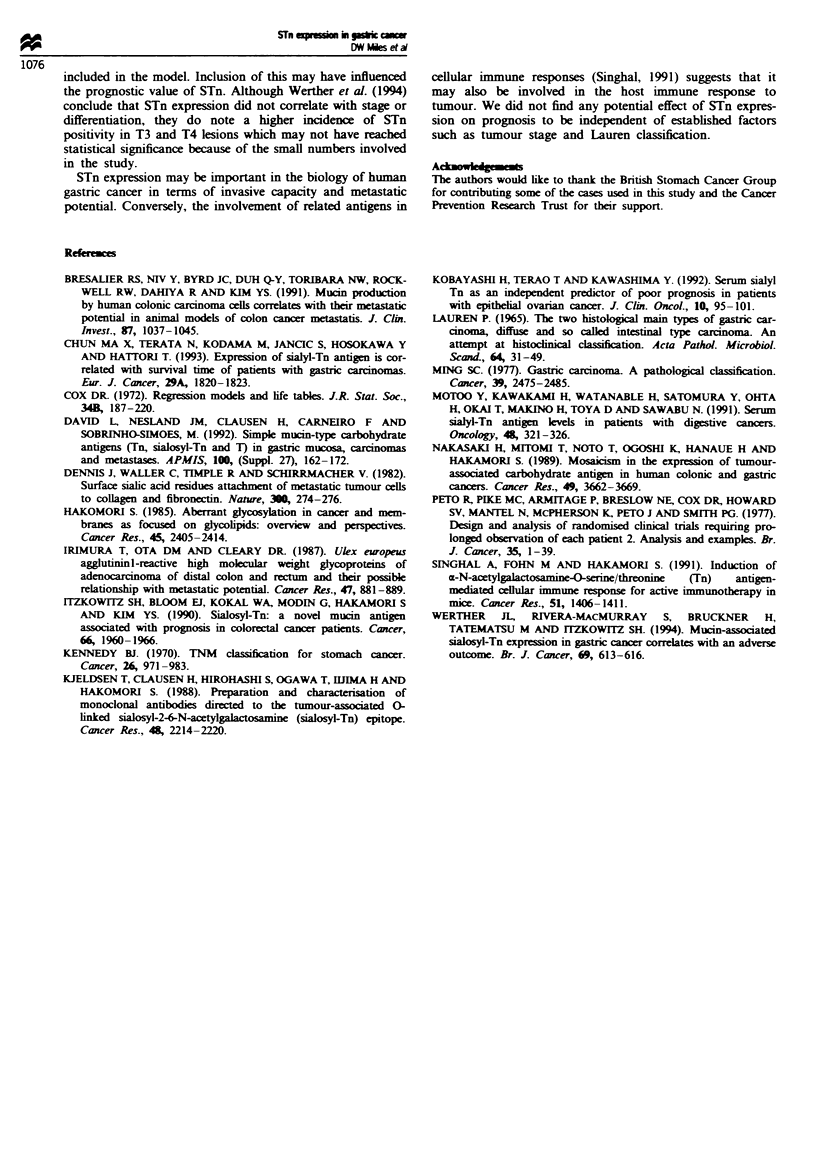# Expression of sialyl-Tn in gastric cancer: correlation with known prognostic factors.

**DOI:** 10.1038/bjc.1995.207

**Published:** 1995-05

**Authors:** D. W. Miles, J. Linehan, P. Smith, I. Filipe

**Affiliations:** Imperial Cancer Research Fund Department of Clinical Oncology, Guy's Hospital, London, UK.

## Abstract

**Images:**


British Journal of Cancer (1995) 71, 1074-1076

© 1995 Stockton Press All rights reserved 0007-0920/95 $12.00

### Expression of sialyl-Tn in gastric cancer: correlation with known prognostic factors

DW Miles[1], J Linehan[2], P Smith[1] and I Filipe[2]

[1]*Imperial Cancer Research Fund Department of Clinical Oncology, Guy's Hospital and* [2]*UMDS Guy's Campus, St Thomas' Street, London SE1 9RT, UK.*

**Summary** Sialyl-Tn (STn) is a core region carcinoma-associated carbohydrate determinant expressed on cancer-associated mucins. Expression of STn has been associated with poor prognosis in colon and ovarian cancer, independent of other prognostic factors such as tumour grade, stage or histological type. Recent studies have suggested that STn expression may be an independent prognostic variable in gastric cancer. We have examined 158 patients with gastric cancer using the antibody B72.3 (Biomira, Edmonton, Alberta, Canada). Of these, 110 patients (70%) expressed STn. Expression of STn did not correlate with tumour differentiation or the Ming classification, but expression was noted more frequently in the relatively good prognosis intestinal type of tumours ($\chi^2 = 6.9$, $P = 0.03$). Conversely, early-stage cancers showed a significantly lower frequency of expression than more advanced cases ($\chi^2 = 13.75$, $P = 0.003$). In this patient group, STn expression did not influence survival, and in multivariate regression analysis only tumour stage and Lauren classification were found to be independent prognostic variables.

**Keywords:** gastric carcinoma; Sialyl-Tn; prognosis

Abnormal glycosylation patterns have been recognised as a feature of carcinoma-associated mucins (Hakomori, 1985). Sialyl-Tn (α[2-6]-*N*-acetylgalactosamine, STn) is a core region carbohydrate antigen of tumour-associated mucin (Kjeldsen *et al.*, 1988) formed by the premature 2-6 sialation of *N*-acetylgalactosamine (Nakasaki *et al.*, 1989). STn expression has been studied in several tumour types and found to be of prognostic significance in colonic carcinoma (Itzkowitz *et al.*, 1990). Circulating antigen has also been detected in gastrointestinal and ovarian malignancies, and raised levels have also been shown to be associated with a poor prognosis (Motoo *et al.*, 1991; Kobayashi *et al.*, 1992). Recent reports have suggested that the expression of STn in gastric carcinoma is an independent prognostic feature (Chun Ma *et al.*, 1993; Werther *et al.*, 1994). In one of the largest published series, we have examined the effect of STn expression on prognosis and its relationship with known prognostic factors.

### Materials and methods

### *Clinical material*

Pathological material from 158 patients whose gastric cancer was considered surgically resectable was examined. Tissue, which was fixed in formalin and embedded in paraffin, was collected between 1979 and 1989 with a median follow-up for the cases studied of 6.9 years. The tumours were classified into intestinal or diffuse (Lauren, 1965) and expanding or infiltrating (Ming, 1977). Stage was defined according to the TNM system (Kennedy, 1970). Survival was known in 139 cases.

### *Immunohistochemistry*

Staining for STn was assessed on 3 μm sections cut from formalin-fixed, paraffin-embedded tissue. Sections were incubated for 60 min at room temperature with the antibody B72.3 (Biomira, Edmonton, Alberta, Canada) raised against ovine submaxillary mucin, and used at a dilution of 1:100. Sections were then treated with biotinylated rabbit anti-mouse immunoglobulin followed by avidin-biotin complex. Peroxidase activity was demonstrated using diaminobenzidine solution and nuclei were counterstained with Mayer's haematoxylin. Negative controls were carried out by substituting buffer for the primary antibody. All cases with stained cells were considered as positive. Immunoreactivity was further characterised as focal (up to 30% of cells positive), patchy (between 30 and 60% of cells positive) and extensive (more than 60% cells positive).

### *Statistical analysis*

Relationships between variables were examined using the chi-squared test. Relapse-free and overall survival were calculated using the method of Kaplan and Meier (Peto *et al.*, 1977) and differences between curves were analysed by the log-rank test. Multivariate analysis was by Cox's proportional hazards model (Cox, 1972).

### Results

STn staining was detected in the cell membrane, luminal mucus and cytoplasm, but its distribution varied between tumours. Membrane staining was a prominent feature in intestinal, well-differentiated tumours (Figure 1), while in

**Figure 1** Intestinal-type adenocarcinoma: STn expression in the cell membrane (immunoperoxidase, bar = 80 μm).

Correspondence: DW Miles
Received 26 August 1994; revised 15 December 1994; accepted 15 December 1994

diffuse signet ring tumours STn staining was predominantly cytoplasmic (Figure 2). An example of lymphatic invasion by an STn-positive tumour is illustrated in Figure 3. Of 158 cases studied, 110 (70%) expressed STn. Of these, 51 cases (46%) showed a focal staining pattern with 15 (14%) and 44 (40%) showing patchy and extensive staining patterns respectively. The clinicopathological features of patients according to STn staining are shown in Table I. Although expression of STn did not correlate with tumour differentiation or the Ming classification, a significantly higher proportion of the intestinal carcinomas expressed STn ($\chi^2$ 6.9, $P = 0.03$). Early-stage cancers showed significantly lower frequencies of STn expression than more advanced cases ($\chi^2 = 13.75$, $P = 0.003$).

**Figure 2** Diffuse signet ring adenocarcinoma: STn expression in the cytoplasm (immunoperoxidase, bar = 50 $\mu$m).

**Figure 3** Lymphatic permeation by STn positive tumour (immunoperoxidase, bar = 50 $\mu$m).

**Table I** Clinical/pathological features of patients by STn staining

| *Factor* | *STn −ve (32%)* | *STn +ve (68%)* | |
|---|---|---|---|
| Stage | | | |
| 1 | 1 (2%) | 8 (8%) | $\chi^2 = 13.75$ |
| 2 | 14 (29%) | 8 (8%) | $P = 0.003$ |
| 3 | 21 (44%) | 62 (63%) | |
| 4 | 12 (25%) | 21 (21%) | |
| Differentiation | | | |
| Good | 10 (21%) | 28 (25%) | $\chi^2 = 5.35$ |
| Moderate | 10 (21%) | 39 (35%) | $P = 0.07$ |
| Poor | 28 (58%) | 43 (39%) | |
| Lauren classification | | | |
| Intestinal | 23 (48%) | 74 (69%) | $\chi^2 = 6.9$ |
| Diffuse | 22 (46%) | 27 (25%) | $P = 0.03$ |
| Mixed | 3 (6%) | 6 (6%) | |
| Ming classification | | | |
| Expanding | 22 (48%) | 57 (56%) | $\chi^2 = 0.53$ |
| Infiltrating | 24 (52%) | 45 (44%) | $P = 0.46$ |

Overall survival of patients by STn staining is illustrated in Figure 4. STn expression did not influence survival in this patient group. In multivariate regression analysis, only tumour stage and Lauren classification were found to be independent prognostic variables (Table II).

### Discussion

Malignant transformation of epithelial cells is frequently associated with altered glycosylation of cell membrane glycoproteins or glycolipids. In the case of *O*-linked oligosaccharides, the premature 2–6 sialation of the Tn antigen (GalNAc) leads to accumulation of the STn antigen (Nakasaki *et al.*, 1989). Expression of this carcinoma-associated antigen in tissue and serum has been shown to be a prognostic factor in colorectal and ovarian adenocarcinomas (Itzkowitz *et al.*, 1990; Kobayashi *et al.*, 1992). It has been demonstrated that tumour-associated glycoproteins (Irimura *et al.*, 1987) and sialic acid residues (Dennis *et al.*, 1982) may be involved in cell–cell or cell–matrix interactions. It has been postulated therefore that the association of STn expression and poor prognosis may be due to such interactions in a manner analogous to the interaction between ELAM-1 and the blood group-related antigens sialyl Lewis A and sialyl Lewis X. Cells expressing STn might therefore be expected to have higher metastatic potential, and this hypothesis is supported by animal data (Bresalier *et al.*, 1991).

Recent data have suggested that STn expression in gastric cancer may be unrelated to established pathological characteristics (David *et al.*, 1992) and may indeed be an independent prognostic factor (Chun Ma *et al.*, 1993; Werther *et al.*, 1994). In the present study using the antibody B72.3, we have demonstrated expression of STn in 110 of 158 cases studied (70%), an incidence of positivity which is in agreement with previous reports. As in the study of Chun Ma *et al.*, we noted a strong correlation between STn positivity and tumour stage. Conversely, however, we have noted a significantly higher incidence of STn expression in the relatively good prognosis intestinal type of tumours (Lauren classification). STn expression was of no prognostic value considering the group as a whole, and in a Cox model only tumour stage and Lauren classification were of independent prognostic value. Although, using a proportional hazards model, Chun Ma concluded that STn expression was an independent prognostic factor ($P = 0.0285$), tumour size (significantly higher in STn-positive tumours) was not

**Figure 4** Overall survival by STn staining.

**Table II** Multivariate analysis of prognostic factors for overall survival

| *Variable* | $\chi^2$ | P-*value* |
|---|---|---|
| Stage | 17.49 | <0.0001 |
| Lauren classification | 7.56 | 0.006 |
| STn staining | 0.87 | 0.3514 |

included in the model. Inclusion of this may have influenced the prognostic value of STn. Although Werther *et al.* (1994) conclude that STn expression did not correlate with stage or differentiation, they do note a higher incidence of STn positivity in T3 and T4 lesions which may not have reached statistical significance because of the small numbers involved in the study.

STn expression may be important in the biology of human gastric cancer in terms of invasive capacity and metastatic potential. Conversely, the involvement of related antigens in cellular immune responses (Singhal, 1991) suggests that it may also be involved in the host immune response to tumour. We did not find any potential effect of STn expression on prognosis to be independent of established factors such as tumour stage and Lauren classification.

**Acknowledgements**

The authors would like to thank the British Stomach Cancer Group for contributing some of the cases used in this study and the Cancer Prevention Research Trust for their support.

### References

BRESALIER RS, NIV Y, BYRD JC, DUH Q-Y, TORIBARA NW, ROCKWELL RW, DAHIYA R AND KIM YS. (1991). Mucin production by human colonic carcinoma cells correlates with their metastatic potential in animal models of colon cancer metastatis. *J. Clin. Invest.*, **87**, 1037-1045.

CHUN MA X, TERATA N, KODAMA M, JANCIC S, HOSOKAWA Y AND HATTORI T. (1993). Expression of sialyl-Tn antigen is correlated with survival time of patients with gastric carcinomas. *Eur. J. Cancer*, **29A**, 1820-1823.

COX DR. (1972). Regression models and life tables. *J.R. Stat. Soc.*, **34B**, 187-220.

DAVID L, NESLAND JM, CLAUSEN H, CARNEIRO F AND SOBRINHO-SIMOES, M. (1992). Simple mucin-type carbohydrate antigens (Tn, sialosyl-Tn and T) in gastric mucosa, carcinomas and metastases. *APMIS*, **100**, (Suppl. 27), 162-172.

DENNIS J, WALLER C, TIMPLE R AND SCHIRRMACHER V. (1982). Surface sialic acid residues attachment of metastatic tumour cells to collagen and fibronectin. *Nature*, **300**, 274-276.

HAKOMORI S. (1985). Aberrant glycosylation in cancer and membranes as focused on glycolipids: overview and perspectives. *Cancer Res.*, **45**, 2405-2414.

IRIMURA T, OTA DM AND CLEARY DR. (1987). *Ulex europeus* agglutinin1-reactive high molecular weight glycoproteins of adenocarcinoma of distal colon and rectum and their possible relationship with metastatic potential. *Cancer Res.*, **47**, 881-889.

ITZKOWITZ SH, BLOOM EJ, KOKAL WA, MODIN G, HAKAMORI S AND KIM YS. (1990). Sialosyl-Tn: a novel mucin antigen associated with prognosis in colorectal cancer patients. *Cancer*, **66**, 1960-1966.

KENNEDY BJ. (1970). TNM classification for stomach cancer. *Cancer*, **26**, 971-983.

KJELDSEN T, CLAUSEN H, HIROHASHI S, OGAWA T, IIJIMA H AND HAKOMORI S. (1988). Preparation and characterisation of monoclonal antibodies directed to the tumour-associated O-linked sialosyl-2-6-N-acetylgalactosamine (sialosyl-Tn) epitope. *Cancer Res.*, **48**, 2214-2220.

KOBAYASHI H, TERAO T AND KAWASHIMA Y. (1992). Serum sialyl Tn as an independent predictor of poor prognosis in patients with epithelial ovarian cancer. *J. Clin. Oncol.*, **10**, 95-101.

LAUREN P. (1965). The two histological main types of gastric carcinoma, diffuse and so called intestinal type carcinoma. An attempt at histoclinical classification. *Acta Pathol. Microbiol. Scand.*, **64**, 31-49.

MING SC. (1977). Gastric carcinoma. A pathological classification. *Cancer*, **39**, 2475-2485.

MOTOO Y, KAWAKAMI H, WATANABLE H, SATOMURA Y, OHTA H, OKAI T, MAKINO H, TOYA D AND SAWABU N. (1991). Serum sialyl-Tn antigen levels in patients with digestive cancers. *Oncology*, **48**, 321-326.

NAKASAKI H, MITOMI T, NOTO T, OGOSHI K, HANAUE H AND HAKAMORI S. (1989). Mosaicism in the expression of tumour-associated carbohydrate antigen in human colonic and gastric cancers. *Cancer Res.*, **49**, 3662-3669.

PETO R, PIKE MC, ARMITAGE P, BRESLOW NE, COX DR, HOWARD SV, MANTEL N, MCPHERSON K, PETO J AND SMITH PG. (1977). Design and analysis of randomised clinical trials requiring prolonged observation of each patient 2. Analysis and examples. *Br. J. Cancer*, **35**, 1-39.

SINGHAL A, FOHN M AND HAKAMORI S. (1991). Induction of α-N-acetylgalactosamine-O-serine/threonine (Tn) antigen-mediated cellular immune response for active immunotherapy in mice. *Cancer Res.*, **51**, 1406-1411.

WERTHER JL, RIVERA-MACMURRAY S, BRUCKNER H, TATEMATSU M AND ITZKOWITZ SH. (1994). Mucin-associated sialosyl-Tn expression in gastric cancer correlates with an adverse outcome. *Br. J. Cancer*, **69**, 613-616.